# *Aspergillus*-superinfected pulmonary metastases following treatment of recurrent endometrial cancer with immune checkpoint inhibitor

**DOI:** 10.1007/s00404-024-07771-0

**Published:** 2024-10-18

**Authors:** Hillary Chappus-McCendie, Shannon Salvador, Gabriel Levin

**Affiliations:** 1 Faculty of Medicine and Health Sciences, McGill University, Montreal, QC Canada; 2 Department of Gynecologic Oncology, Jewish General Hospital, McGill University, Montreal, QC Canada

**Fig. 1.**
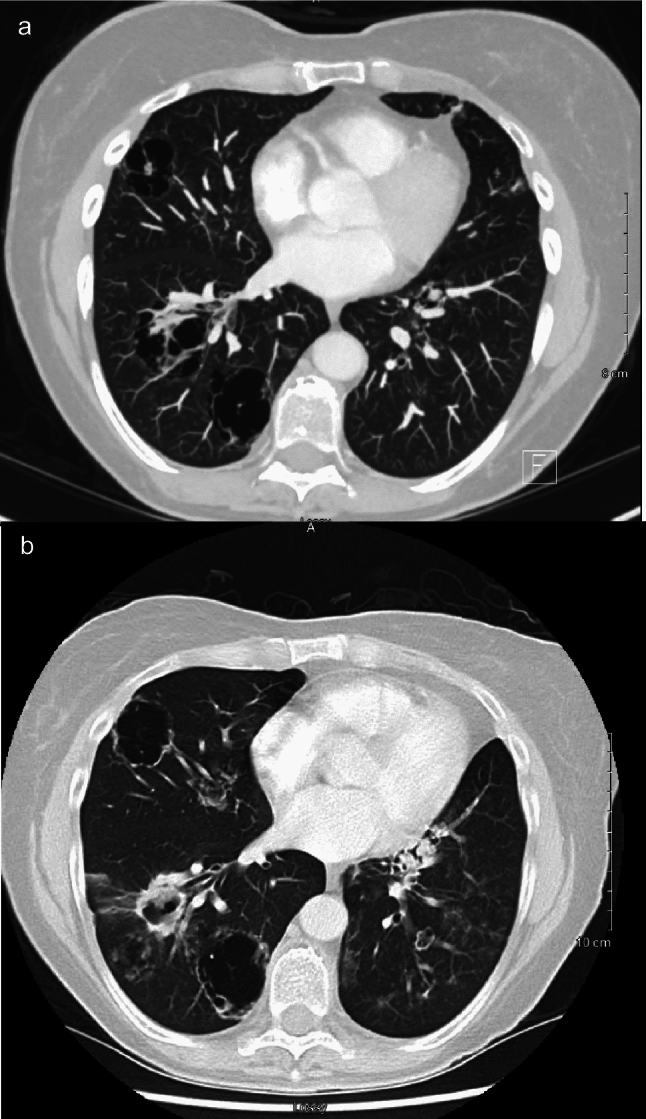
Computed tomography scan of a 58-year-old patient’s chest on initial presentation (**a**) and three months later (**b**). The scan on initial presentation demonstrates cystic masses in the lungs. The scan three months later depicts metastatic disease with ongoing superimposed invasive aspergillosis.

A 58-year-old woman who was known for recurrent endometrial cancer (EC) with pulmonary metastases who was on treatment with lenvatinib and pembrolizumab. Over the course of her treatments, she had developed a chronic cough with upper back pain. Following the sixth lenvatinib and pembrolizumab treatment, a computed tomography (CT) scan showed several large cystic masses in both lungs (Fig. 1a). These lesions were concerning for cavitary metastases with a differential diagnosis of possible infected cysts in the setting of cystic lung disease. Following her seventh treatment cycle, the patient continued to have progressive worsening of her cough with hemoptysis, although she was not neutropenic, nor did she receive corticosteroids. A percutaneous lung biopsy of the left upper lobe lesions was positive for *Aspergillus*. The patient began treatment with voriconazole and continued to be followed in the gynecologic oncology clinic. A follow-up CT scan three months later revealed findings consistent with progressive metastatic lung disease with ongoing superimposed invasive aspergillosis (Fig. 1b). A second lung biopsy was performed, which confirmed the presence of EC in the lungs.

Aspergillosis is a spectrum of diseases, caused by the fungus *Aspergillus,* which are more common in neutropenic or immunosuppressed patients [1].

The use of lenvatinib and pembrolizumab has proven to be effective at increasing length of progression-free survival and overall survival in previously treated advanced EC [2]. A previous study reported two cases of invasive pulmonary aspergillosis, none of which were in patients receiving pembrolizumab [3]. To our knowledge, this is the first reported case of *Aspergillus*-superinfected pulmonary metastases in a patient undergoing combined lenvatinib and pembrolizumab therapy for EC. Given the high mortality of such infections, invasive aspergillosis, although uncommon, should be considered a differential diagnosis in patients presenting with respiratory symptoms following treatment with immune checkpoint inhibitors.
